# STEMI in a Young Male after Use of Synephrine-Containing Dietary Supplement

**DOI:** 10.1155/2018/7074104

**Published:** 2018-04-01

**Authors:** Dileep Unnikrishnan, Radhika Annam, Aasems Jacob, Braghadheeswar Thyagarajan, Peter Farrugia

**Affiliations:** Department of Internal Medicine, Monmouth Medical Center, Long Branch, NJ, USA

## Abstract

A twenty-two-year-old male with no significant past medical history who presented with chest pain was found to have ST-segment elevation in leads II, III, aVF, and V4–V6. On subsequent EKGs, patient had new ST-segment elevations in anterolateral leads with dynamic changes. Cardiac catheterization showed acute dissection with thrombosis of the distal left main coronary artery leading into the ostial left anterior descending artery. The patient had no cardiac risk factors including hypertension, hyperlipidemia, diabetes, or family history of early cardiac disease. On further inquiry, the patient was found to be on two separate performance-enhancing supplements which contained synephrine, a sympathomimetic chemical which was later attributed as the cause of his acute coronary syndrome. Synephrine acts on alpha-1 adrenergic receptors causing peripheral and coronary vasoconstriction, hypertension, and hyperglycemia. Increased hemodynamic stress on the coronary arteries can lead to fatal dissections. Ours is an atypical case of synephrine-induced nonatherosclerotic spontaneous coronary artery dissection which helps caution the physicians about the importance of dietary supplement use in the history and possible side effects of such performance-enhancing additives.

## 1. Introduction

Several weight-loss and performance-enhancing dietary supplements are available over the counter in the US markets. Adding sympathomimetic chemicals like ephedra-containing compounds are highly effective in performance enhancement with the potential of causing fatal cardiac and respiratory side effects. Ephedra-containing compounds were banned by the FDA. However, this has led to the use of alternative sympathomimetic compounds like synephrine in these dietary supplements [1]. These selective alpha blockers can potentially be dangerous due to their effects like elevated systemic blood pressure, coronary vasospasm, and arterial dissections. Nonatherosclerotic spontaneous coronary artery dissection (NA-SCAD) is a rare cause of nontraumatic coronary dissection in young adults due to weakening of the arterial wall from hemodynamic stress. Most commonly associated factors include fibromuscular dysplasia, postpartum status, connective tissue disorders, and hormonal therapy [2]. Here, we report a case of a massive STEMI in a young African American male secondary to the use of synephrine-containing dietary supplement. On review of literature, we were able to find only one other similar case of young STEMI attributed to sympathomimetic dietary supplement administration [3].

## 2. Case Presentation

**Figure 1:**
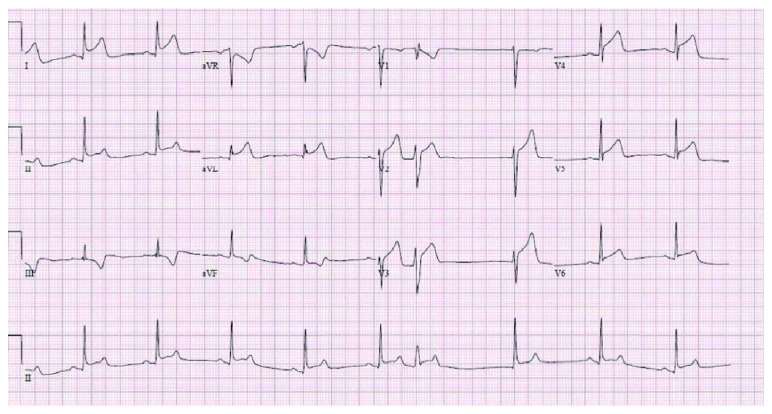
EKG of the patient showing ST elevation in leads I, avL, II, III, and V2–V6.

A 22-year-old African American male presented to the ER with acute onset of “pressure-like” substernal, nonradiating chest pain. His symptoms started while playing basketball. The pain was associated with shortness of breath and nausea. He had no significant past medical history or family history of coronary artery disease or sudden deaths. He denied excessive caffeine use, smoking, or alcohol intake. There was no reported use of any illicit drugs such as cocaine or amphetamines and was physically very active. Vital signs on admission included blood pressure of 138/89 mmHg, heart rate of 94/min, and respiratory rate of 22 breaths/min. His BMI was 28. Physical examination was unremarkable with no chest tenderness on palpation and no jugular venous distention. Heart sounds were normal with no murmurs or additional sounds. The electrocardiogram showed ST-segment elevation myocardial infarction (STEMI) in leads II, III, aVF, and V4–V6 (Figure 1). On subsequent EKGs, the patient had new ST-segment elevations in anterolateral leads with dynamic changes. Laboratory studies were pertinent for a troponin-I level of 0.83 ng/ml. The patient was given aspirin and ticagrelor loading doses, metoprolol succinate 25 mg, intravenous morphine, intravenous heparin drip, and sublingual nitroglycerin which did not relieve his chest pain.

**Figure 2:**
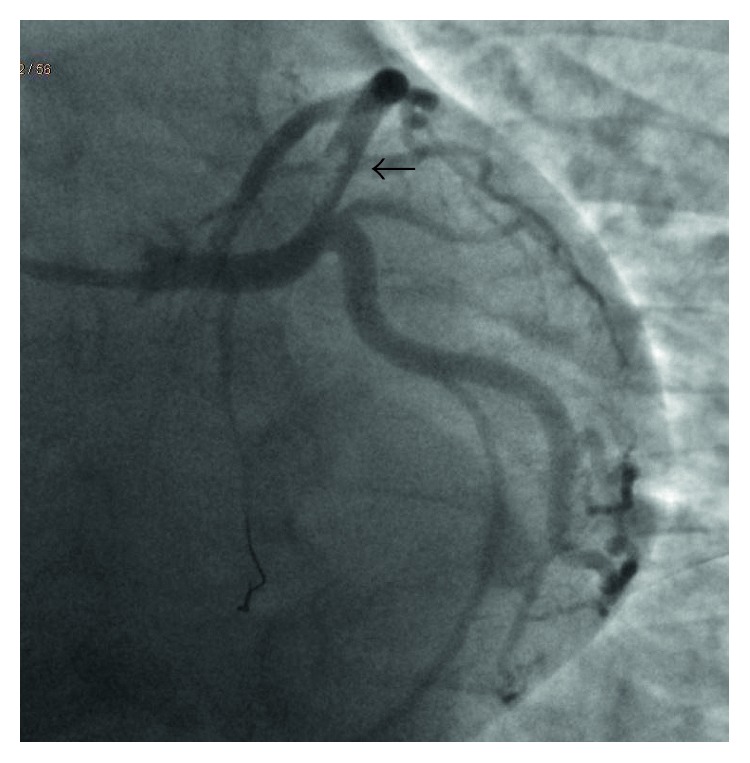
Coronary angiogram: left anterior oblique view of LAD prior to percutaneous coronary intervention showing dissection and clot in the proximal LAD (black arrow).

**Figure 3:**
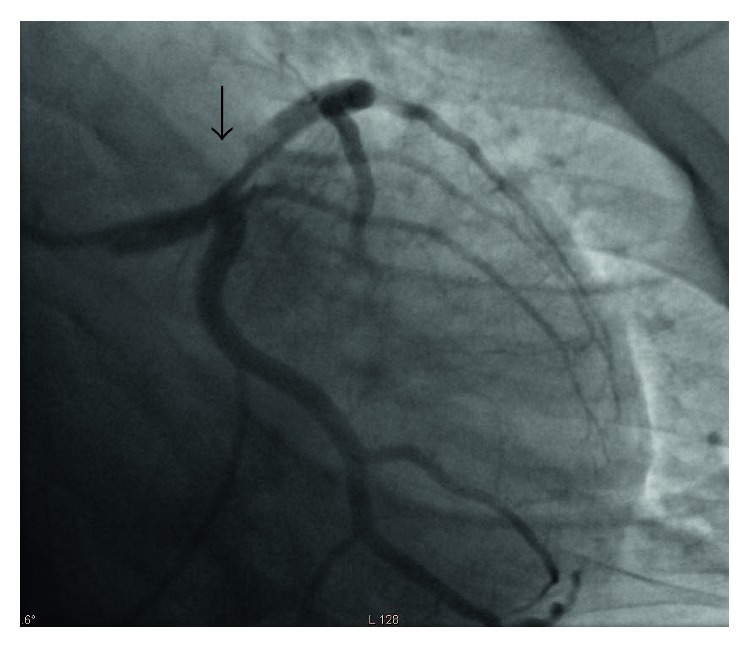
Coronary angiogram: right anterior oblique view of LAD prior to percutaneous coronary intervention showing dissection with clot in proximal LAD with TIMI 2 flow distally (black arrow).

**Figure 4:**
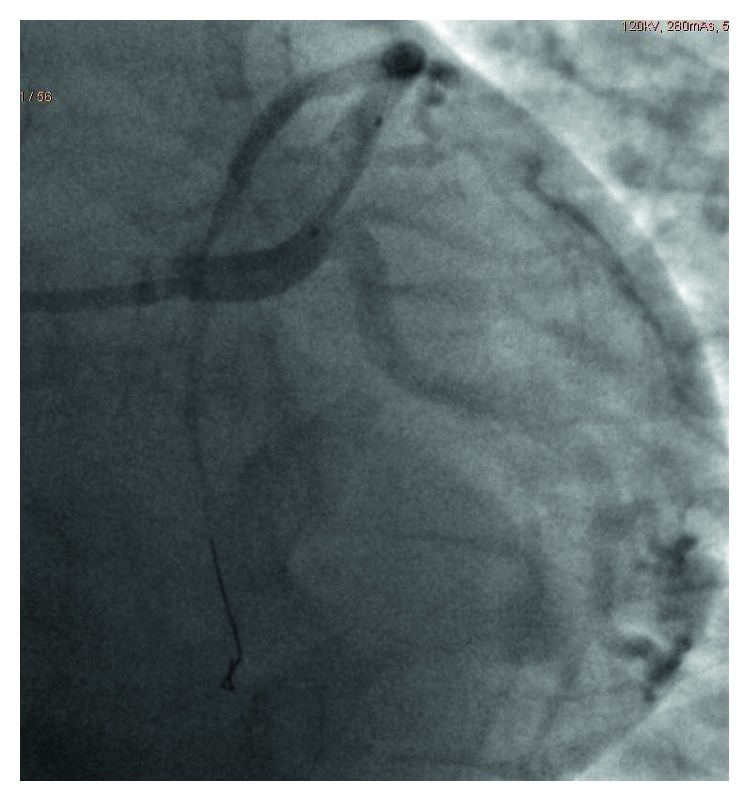
Coronary angiogram: left anterior oblique view of LAD during intervention with bare metal stent in the proximal LAD.

**Figure 5:**
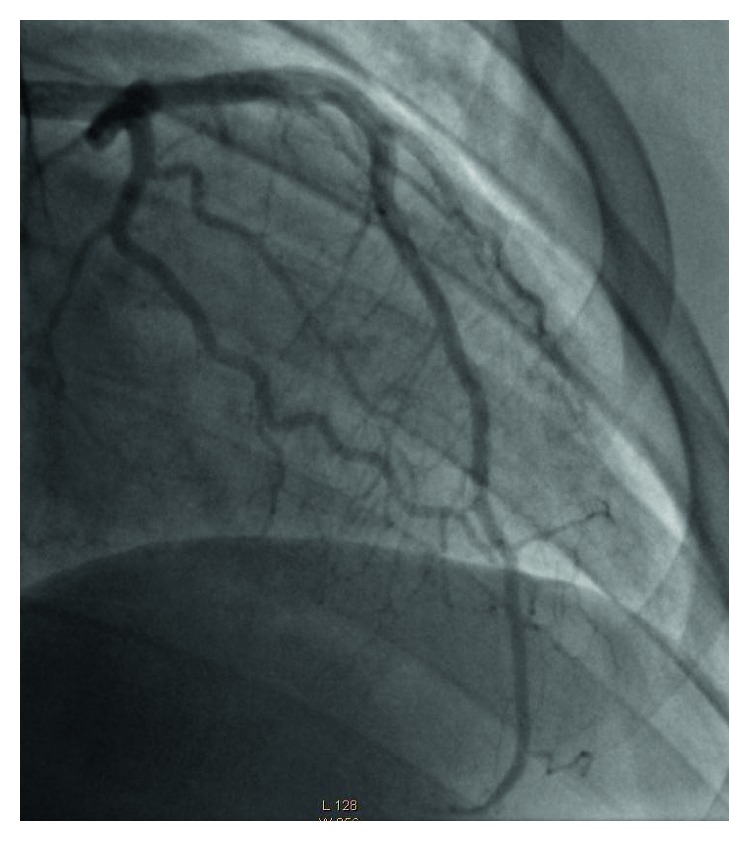
Coronary angiogram: right anterior oblique view of LAD after intervention showing bare metal stent in the proximal LAD with TIMI 4 flow distally.

The patient was taken for emergent cardiac angiography within 60 minutes of presentation and revealed acute dissection with thrombosis of the distal left main coronary artery (LCA) leading into the proximal left anterior descending artery (LAD) (Figures 2 and 3). An EBU 3.5 guide catheter was advanced over the wire with repeat injection of the left main performed revealing persistence of acute occlusion. Repeat angiography after balloon inflation revealed significant improvement in flow from TIMI 2 to TIMI 4. An extending thrombus was noted in the distal LAD which was suctioned out. Following this, a bare metal stent was inserted in the distal LCA ostial and the proximal LAD (Figures 4 and 5).

After catheterization, the patient was pain-free and hemodynamically stable. He was on epitifibatide infusion for a total of 18 hours. Other medications including carvedilol, lisinopril, aspirin, ticagrelor, and atorvastatin were initiated. The patient's peak troponin went up to 48.56 ng/ml in less than 24 hrs of initial laboratory studies and subsequently was trending down. Additional laboratory studies including hemoglobin A1c and lipid profile were normal. He was also worked up for thrombotic conditions which might lead to arterial thrombi such as homocystinemia and antiphospholipid antibody syndrome both of which were negative. Transthoracic echocardiography performed revealed preserved left ventricular systolic function normal with ejection fraction estimated to be 0.50 to 0.54%. There was no patent foramen ovale that might have led to paradoxical embolus. A urine and serum toxicology screen was also done on the patient who came back as negative for cocaine or methamphetamine.

After the procedure, the patient was further questioned to elicit any history that might shine light to the etiology of his STEMI. He admitted to the use of two different weight-loss products (Performix™ stim-free; Performix SST) for the past 1 year, taking one capsule up to three times a day as indicated on the product label. However, he took Performix SST for the first time on the afternoon of presentation, drinking 3 scoops mixed with water, approximately 2 hours before started playing basketball. On reviewing, the contents of the supplement, it was noted to contain synephrine, an epinephrine analogue. Other contents of the mixture consisted of anhydrous caffeine, sensoril ashwagandha extract, bitter orange extract, n-methyltyramine, hordenine, tyramine, octopamine, huperzine A, yohimbine HCL, mucuna pruriens, and Bioperine. The patient was advised to avoid the use of the supplements. He was discharged home from the hospital with outpatient cardiology follow-up and advice to be maintained on dual antiplatelet therapy for at least one year.

## 3. Discussion

This is an interesting case of a young active male with no apparent past medical or familial risk factors who presented with STEMI secondary to dissection with thrombosis of distal left main coronary artery extending to proximal LAD. The patient's condition could be attributed to his use of synephrine-containing weight-loss and performance-enhancing supplements. Administration of beta-blockers preprocedure might also have led to the unimpeded alpha action of the adrenergics in this case. Synephrine is a naturally occurring alkaloid which has been used in combination with caffeine in several weight-loss and dietary supplements. Pharmacologically synephrine has its action on alpha-1 adrenergic receptors causing effects such as peripheral vasoconstriction, hypertension, and hyperglycemia [4]. Unsupervised use of such supplements could be risky and even fatal. In addition, mixing several stimulants and natural compounds such as caffeine and bitter orange extract with synephrine can lead to increase in the adverse effects profile of each [5]. A definitive causal relation between the dietary supplement intake and coronary dissection cannot be drawn in this case; however, given the paucity of any other likely risk factors, the event is presumably secondary to the hypertension and sympathetic overactivity from synephrine. Dietary supplement and weight reduction drug industries are highly lucrative, and the unscrupulous and potentially dangerous practice of adding sympathomimetic compounds for quick results is rampant [6]. More FDA regulations have to be implemented in this regard for the safety of the users. For any such patients presenting with acute coronary syndrome, the use of beta-blockers should be avoided [7]. Furthermore, it is important for physicians to be cognizant of sympathomimetics in the dietary supplements and elicit such history in atypical cases such as the one presented here in order to avoid diagnostic confusion and inadvertent harm to the patient.
